# Regulation of Intestinal Stem Cell Stemness by the Aryl Hydrocarbon Receptor and Its Ligands

**DOI:** 10.3389/fimmu.2021.638725

**Published:** 2021-03-10

**Authors:** Paul J. Wisniewski, Mitzi Nagarkatti, Prakash S. Nagarkatti

**Affiliations:** Pathology, Microbiology and Immunology, School of Medicine, University of South Carolina, Columbia, SC, United States

**Keywords:** gut epithelium, intestinal stem cells, aryl hydrocarbon receptor, aryl hydrocarbon receptor ligands, morphogenetic pathways

## Abstract

Maintenance of intestinal homeostasis requires the integration of immunological and molecular processes together with environmental, diet, metabolic and microbial cues. Key to this homeostasis is the proper functioning of epithelial cells originating from intestinal stem cells (ISCs). While local factors and numerous molecular pathways govern the ISC niche, the conduit through which these processes work in concordance is the aryl hydrocarbon receptor (AhR), a ligand-activated transcription factor, whose role in immunoregulation is critical at barrier surfaces. In this review, we discuss how AhR signaling is emerging as one of the critical regulators of molecular pathways involved in epithelial cell renewal. In addition, we examine the putative contribution of specific AhR ligands to ISC stemness and epithelial cell fate.

## Introduction

Maintenance of intestinal homeostasis is governed extensively by the integration of both molecular and immunological processes. This integration is further mediated by the presence of enteric microorganisms that colonize the gastrointestinal (GI) tract. The crosstalk between intestinal microorganisms and the host in which they reside occurs at the gut mucosa, a specialized intestinal tissue that represents one of the body's most important interfaces with the environment. The gut mucosa is comprised of the gut epithelium, a monolayer of epithelial cells that has critical functions in avoiding self-digestion, contending with luminal contents without eliciting overt immune responses and promoting self-tolerance (1). Due to its significance, the gut epithelium demonstrates an astounding renewal capacity as the entire intestinal lining is replenished completely within 5 days (2–4). Homeostasis of the gut epithelium itself is maintained by an intestinal stem cell (ISC) compartment that resides at the base of intestinal crypts, giving rise to specialized epithelial cell lineages (5). As such, these ISCs are crucial for the renewal of the differentiated progeny that comprise the gut epithelium. However, this rapid rate of renewal imposes greater demands on the cellular hierarchy of the gut epithelium as well as a greater risk of developing intestinal malignancies (6). What remains to be explored is the extent to which ISCs can be influenced by environmental factors to maintain or restore intestinal homeostasis.

Of note is the modulation of immune responses from compounds derived from both endogenous and exogenous sources via the aryl hydrocarbon receptor (AhR), a ligand-activated transcription factor that integrates environmental, dietary, microbial and metabolic cues to control transcriptional programs in a ligand-, cell- and context-specific manner (7). While there are some recent reviews on the role of AhR in the regulation of inflammation through induction of anti-inflammatory signaling involving IL-10, IL-22, prostaglandin E_2_, and Foxp3 (8), to the best of our knowledge, there are no reviews on the role of AhR in ISC function and regulation. Using floxed *Ahr* to *Villin*-*Cre* mice, Metidji and colleagues have recently shown AhR expression in intestinal epithelial cells (IECs) to be critical for ISC homeostasis and gut barrier integrity as it plays a dominant role in tempering Wnt signals (9). Expression of tryptophan metabolizing enzyme, indoleamine 2,3-dioxygenase 1 (IDO1), in IECs has also shown to enhance differentiation of secretory cells and mucus production in IEC-specific transgenic mice (mouse line pVil-EGFP/IDO1) challenged with dextran sodium sulfate (DSS), 2,4,6-trinitrobenzene sulfonic acid (TNBS) or enteropathogenic *Escherichia coli* (10). Because induction of IDO depends on AhR expression and kynurenine produced by IDO acts as an AhR agonist, these studies suggested that AhR promotes intestinal homeostasis. Additionally, AhR has shown to sense genotoxic compounds found in the diet and protect stem cells against genotoxic stress through the induction of IL-22 by innate lymphocytes (11). Together, these are a few examples that highlight the extent to which AhR activation mediates the regulation ISC stemness. In this review, we examine current knowledge on how AhR activation can modulate ISC stemness through essential signals of epithelial cell differentiation.

## The Intestinal Stem Cell Compartment

**Figure 1:**
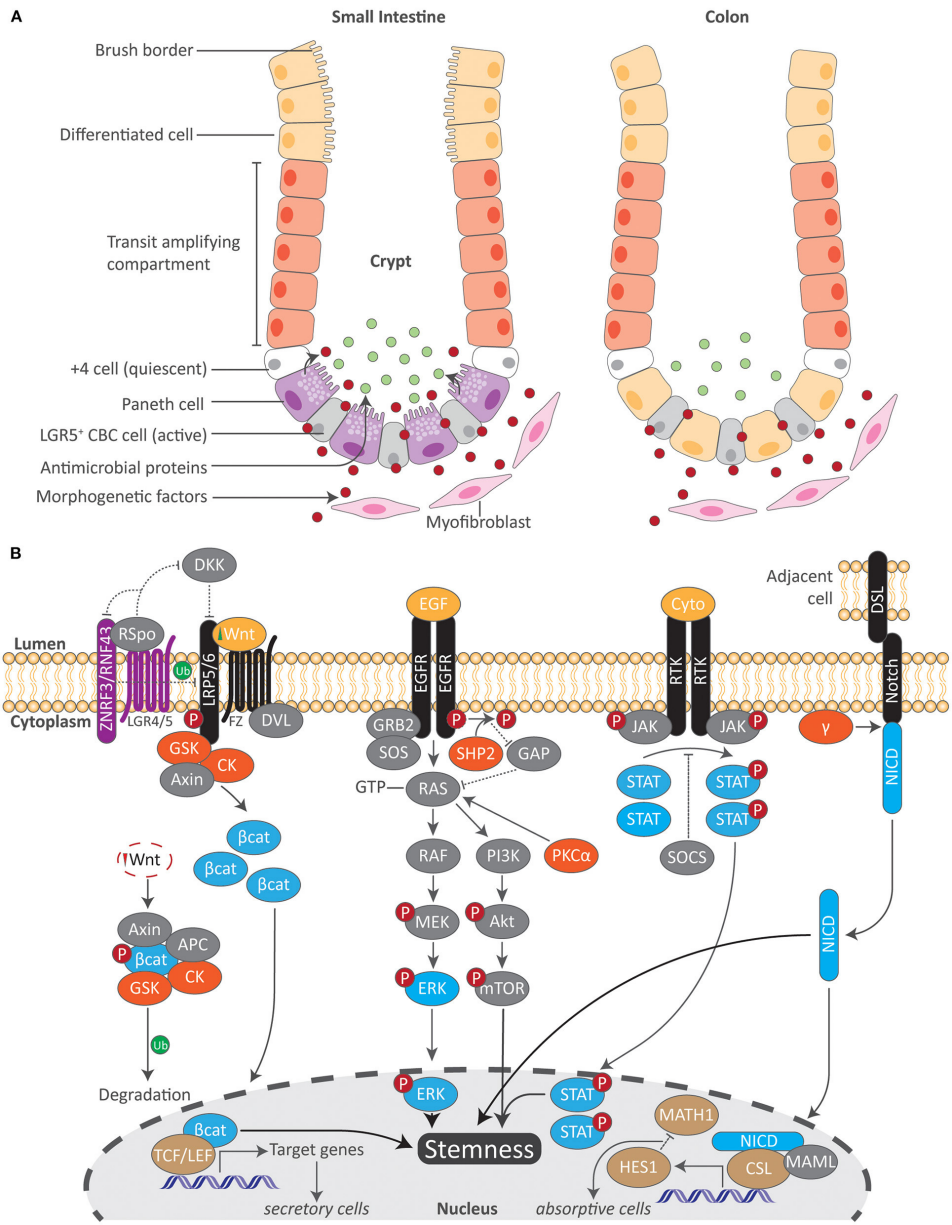
Overview of the intestinal stem cell compartment and molecular cascades related to AhR signaling. **(A)** Active and quiescent stem cells reside at the crypt base in both the small and large intestine. Local morphogenetic factors produced from small intestinal Paneth and subepithelial mesenchymal cells regulate ISC activity. As stimulated ISCs migrate upward into the transit amplifying compartment, they then become committed differentiated cells. A brush border resides at the apical surface of epithelial cells in the small intestine which maximizes the absorptive surface area. In contrast, both Paneth cells and a brush border are absent in the colon. While the absence of Paneth cells in the colon results in decreased antimicrobial proteins, the colon employs other mechanisms to maintain intestinal homeostasis. LGR5 leucine-rich-repeat containing G-protein coupled receptor 5, CBC crypt base columnar. **(B)** Numerous molecular pathways orchestrate epithelial cell fate in concordance with local morphogenetic factors within the ISC. Key proteins in each signaling pathway (blue) interact with transcription factors (brown) to regulate gene transcription. Upstream of this, additional kinases (orange) modulate these pathways. Both Wnt/β-catenin and Notch signaling play a pivotal role in regulating the differentiation of either secretory or absorptive epithelial cell types. Together with EGF/MAPK and JAK/STAT signaling as well as others, these pathways work in tandem to regulate ISC stemness. Akt protein kinase B, APC adenomatous polyposis coli, Bcat beta catenin, CK casein kinase, CSL CBF1/SU/LAG1, Cyto cytokine, DKK Dickkopf, DSL Delta/Serrate/LAG2 transmembrane ligands, DVL Disheveled, EGF epidermal growth factor, EGFR epidermal growth factor receptor, γ gamma secretase, GAP GTPase-activated protein, GRB2 growth factor receptor-bound protein 2, GSK glycogen synthase kinase, HES1 hairy and enhancer of split 1, JAK/STAT janus kinase/signal transducer and activator of transcription, MAML Mastermind-like, MATH1 Protein atonal homolog 1, mTOR mammalian target of rapamycin, NICD Notch intracellular domain, PI3k phosphoinositide 3-kinase, PKCalpha protein kinase c alpha, RNF43 ring finger 43, Rspo respondins, RTK receptor tyrosine kinase, SHP2 Src homology 2 phosphatase 2, SOCS suppressors of cytokine signaling, SOS son of sevenless, TCF/LEF T-cell factor/lymphoid enhancer-binding factor, Ub ubiquination, ZNRF3 zinc and ring finger 3.

While several populations of ISCs have been described, the driving force of epithelial cell renewal and tissue repair are the fast-cycling crypt base columnar (CBC) stem cells marked by a leucine-rich-repeat containing G-protein coupled receptor 5 (LGR5) (2, 12, 13). These ISCs, or LGR5-positive (+) CBC stem cells, divide daily and reside at the crypt base (14) (Figure 1A). Due to the limited space of intestinal crypts, ISCs undergo ‘neutral competition’ in which half are pushed out of the ISC niche at random to the above transit-amplifying (TA) compartment where they then become committed progenitor cells (14, 15). Immediately preceding TA cells is a slow dividing ‘reserve stem cell’ or position 4/ +4 cell population, counting the adjacent cells from the crypt base, that replenishes the pool of active stem cells under normal circumstances or fully differentiates into epithelial cells in the advent of a disrupted LGR5+ compartment such as during acute inflammation (16, 17). What governs this variable stem cell activity and states of competency are niche-derived signals, such as growth factors and wingless-related integration site (Wnt) ligands, from neighboring Paneth cells within the gut epithelium and from subepithelial mesenchymal cells, including the rare winged helix transcription factor Foxl1 expressing (Foxl1+) telocytes which maintain intestinal crypt cell proliferation and promote homeostatic renewal of the gut epithelium, as well as the recently identified CD34+ Gp38+ mesenchymal cells which rapidly respond to intestinal injury and produce a myriad of factors involved in ISC maintenance and tissue repair (18–21). ISCs are therefore subjected to and directed in activity by a host of proximal signals that encompass the ISC niche. What orchestrates the generation of new epithelial cells from ISCs and their subsequent functional specialization in tandem with ISC niche-derived signals are several molecular pathways. Among these are the Wnt/β-catenin, Notch, Hedgehog and bone morphogenic protein (BMP), as well as the epidermal growth factor receptor (EGFR) and ephrin (Eph) pathways which direct ISC proliferation and cell positioning (21). Here, we provide an overview of select signals relevant to AhR activation (Figure 1B).

## AhR Signaling and Regulation

**Figure 2:**
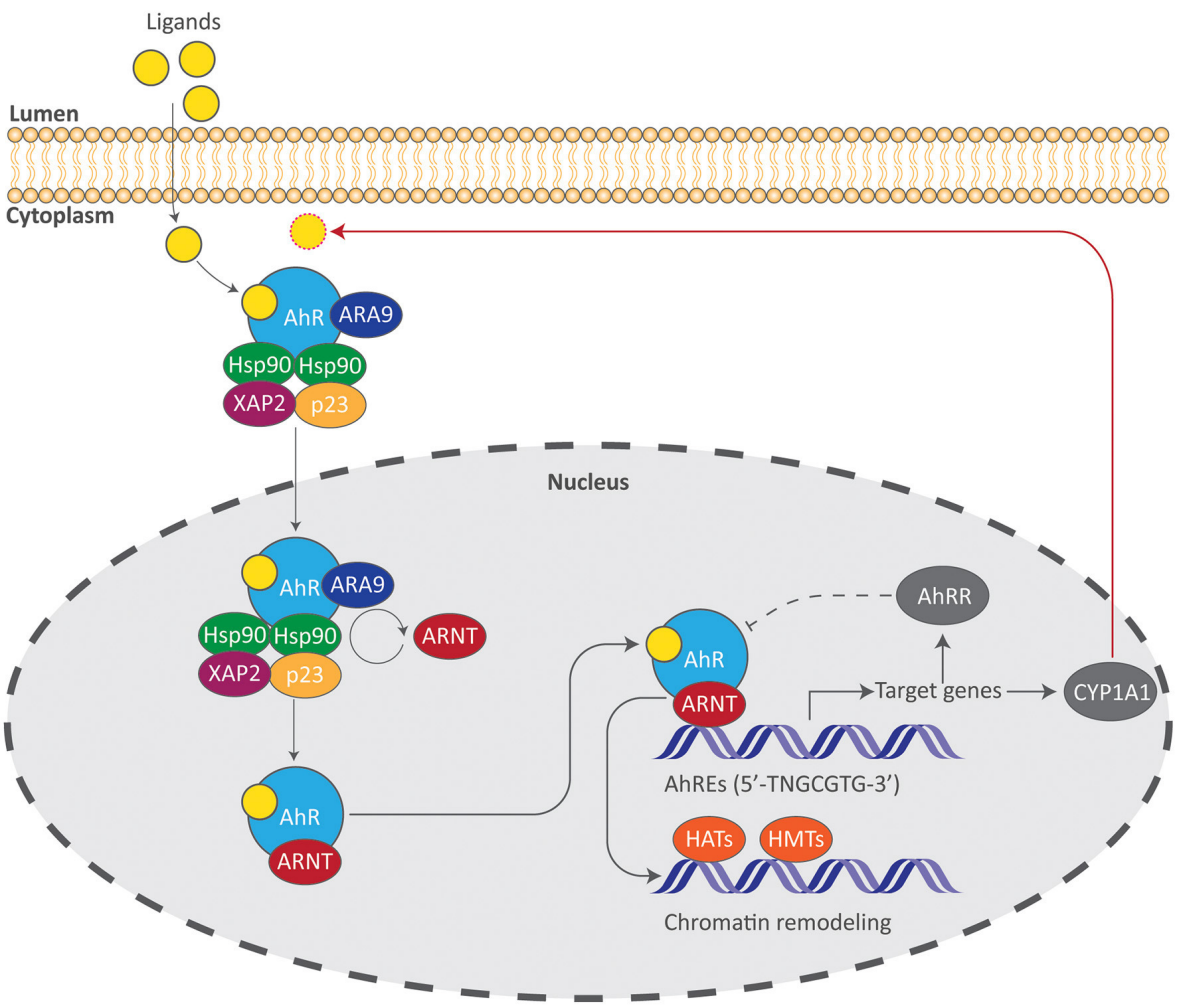
The AhR signaling pathway. Induction of AhR signaling requires binding of a ligand, allowing the AhR complex to translocate into the nucleus and initiate transcriptional events. Gene products such as the AhR repressor protein (AhRR) and the cytochrome P450 enzyme CYP1A1 suppress AhR signaling by acting as a direct antagonist to the AhR (dotted line) or by metabolizing AhR ligands (red arrow). AhREs aryl hydrocarbon responsive elements, ARA9 AhR-associated protein 9, ARNT AhR receptor nuclear translocator, HATs histone acetyltransferases, HMTs histone methyltransferases, Hsp90 heat-shock protein 90, XAP2 X-associated protein 2.

The AhR is a basic helix-loop-helix (bHLH) ligand-dependent transcription factor that responds to a variety of ligands due to its malleable ligand-binding site and is the only member of the bHLH superfamily of transcription factors that can be activated by ligands (22). Signaling of the AhR involves a central PER-ARNT-SIM (PAS) domain that is involved in DNA recognition, ligand binding, and chaperone interactions which are critical for ensuing transcriptional events (Figure 2). In its inactivated form, the AhR resides in the cytoplasm within a chaperone complex comprised of heat-shock protein 90 (Hsp90), p23, X-associated protein 2 (XAP2), and AhR-associated protein 9 (ARA9) (23). Hsp90 preserves a conformational state of the AhR that prevents unsolicited translocation into the nucleus and allows binding of a ligand, while the phosphoprotein p23 facilitates the interaction between the AhR and Hsp90 (24, 25). XAP2 regulates AhR turnover and ARA9 augments AhR signaling by increasing available binding sites and by increasing the amount of cytosolic AhR (26, 27). Upon binding of a ligand, the AhR undergoes structural modifications that expose nuclear localization sequences in which two adjacent protein kinase C sites become phosphorylated (28–30). Once translocated, AhR dissociates from its chaperone complex as AhR receptor nuclear translocator (ARNT) replaces Hsp90 forming a heterodimer (23). This AhR-ARNT heterodimer binds to *cis* elements of DNA that contain aryl hydrocarbon responsive elements (AhREs, also known as xenobiotic- or dioxin-response elements). These regulatory elements containing the core sequence 5'-TNGCGTG-3' can be found in the promoter regions of numerous target genes including cytochrome P450 enzymes such as CYP1A1, which metabolizes AhR ligands, thereby suppressing its activation (31). Once bound to AhREs, this complex acts as a transcriptional complex that can alter transcriptional activity and chromatin structure through histone acetyltransferase and methyltransferase activity (32). AhR activity is tightly controlled by two primary mechanisms in which the first involves proteolytic degradation 4 h after the ligand-bound AhR has associated with AhREs and is then exported from the nucleus (33). The second involves the AhR repressor protein (AhRR) which is structurally analogous to the AhR but does not require a ligand to translocate into the nucleus and interacts with ARNT. It is upregulated upon AhR activation and therefore acts as a transcriptional repressor (34).

## Interaction Between the AhR and Select Molecular Signals of ISC Homeostasis

**Figure 3:**
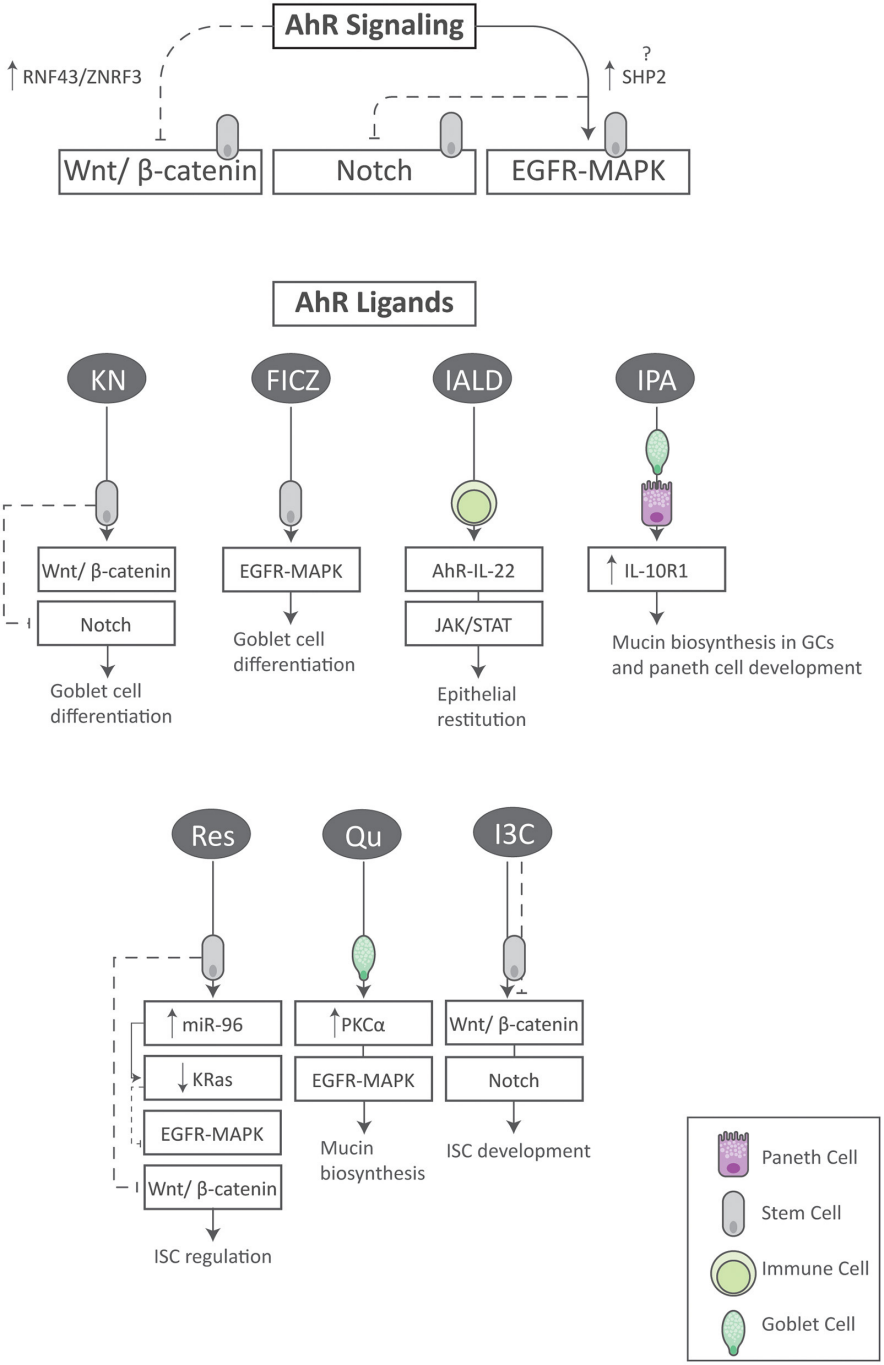
Summary of AhR signaling and its ligands on aspects of ISC homeostasis. Activation of the AhR regulates the Wnt, Notch and EGFR/MAPK signaling pathways in ISCs. In addition, specific AhR ligands synergize with key stemness pathways within various cell types to exert unique and beneficial effects. FICZ tryptophan derivative 6-formylindolo (3, 2-b) carbazole, I3C indole-3-carbinol, IALD indole-3-aldehyde, IL-10R1 interleukin-10 receptor 1, IPA indole-3-propionic acid, GC goblet cell, KN kynurenine, Qu quercetin, Res resveratrol.

At present, an increasing volume of evidence indicates that the AhR is a pleiotropic regulator of molecular processes that extend beyond its historical role as a xenobiotic sensor. In particular is its emerging role in immune development and function at barrier surfaces including the skin, respiratory tract and GI tract (35). In addition, AhR activation may contribute to intestinal homeostasis by regulating ISC stemness and progeny through morphogenetic signals and others as summarized here (Figure 3). As so eloquently defined by Aponte and Caicedo, stemness in this regard combines the ability of ISCs to perpetuate its lineage, to give rise to differentiated epithelial cells, and to interact with its environment to maintain a balance between quiescence, proliferation and regeneration (36).

### Wnt/β-catenin

The canonical Wnt/β-catenin pathway plays a pivotal role in the establishment of tissue architecture during development and in homeostasis of adult tissues (37). In the intestine, it is critical for the proliferation and maintenance of ISCs as well as for the differentiation of goblet cells (GCs) (38, 39). Here, β-catenin is an essential cytoplasmic signal transducer (40). Given its role in the maintenance of intestinal homeostasis, aberrant Wnt/β-catenin signaling has shown to be a hallmark of colorectal cancer (CRC) development characterized by a loss of the tumor suppressor adenomatous polyposis coli (APC) and hyperactivation of Wnt/β-catenin signals (41). In contrast, a putative mechanism by which AhR activation regulates the Wnt/β-catenin pathway has recently been determined to be through the activity of E3 ubiquitin ligases RNF43 and ZNRF3 which target Wnt frizzled receptors for degradation in ISCs, thereby inhibiting Wnt signaling (42). Specifically, selective ablation of the AhR or overexpression of CYP1A1 in IECs potentiated ISC proliferation (as indicated by Ki67 expression) and inflammation-induced tumorigenesis (9). This was accompanied by a reduced RNF43 and ZNRF3 expression, and a concomitant increase in β-catenin and Wnt target gene expression (9). These results show that AhR deficiency or the degradation of its ligands in IECs promotes the hyperactivation of Wnt signaling due to a selective defect in the induction of key negative pathway regulators. In turn, this highlights the potential of physiological AhR signals to temper Wnt responsiveness in ISCs. Using an *in vitro* model of wound healing, Kasai et al. have further shown that disruption of adherens junctions by Spinner Modification (S-MEM) in Caco-2 cells enhances the interaction between β-catenin and AhR but not with TCDD treatment (an AhR agonist) as evidenced by immunoprecipitation and that the ablation of β-catenin by siRNAs enhances the induction of CYP1A1 mRNA with S-MEM or TCDD treatment (43). This suggests that while ablation of AhR may potentiate Wnt signals, ablation of β-catenin may then potentiate AhR signals in response to tissue injury though direct degradation of β-catenin by ligand-activation of the AhR remains questionable. As recently demonstrated, only one primary association between β-catenin and the AhR *in vitro* could be found after failing to induce β-catenin degradation by AhR activation with various AhR ligands in multiple cell lines, that β-catenin does enhance AhR-mediated transcriptional activation (44). As such, the most significant discrepancy is whether the above interaction is assessed *in vivo* or *in vitro*. Consequently and as the findings of Kasai et al. suggest, that despite TCDD not enhancing the interaction between β-catenin and the AhR, local factors following tissue injury may act as endogenous AhR ligands to then temper Wnt signals which could explain the hyperactivation of Wnt signals *in vivo* following IEC-specific ablation of AhR.

### EGFR-MAPK/ERK and Notch

Epidermal growth factor (EGF) is an extracellular ligand produced from neighboring Paneth and subepithelial mesenchymal cells that plays a pivotal role in intestinal growth as it potentiates cell survival and ISC proliferation through downstream Mitogen-activated protein kinase (MAPK) signals (45–47). MAPKs are vital signaling molecules that influence a broad range of cellular processes including proliferation and differentiation in IECs (47). Together with the Wnt/β-catenin pathway, MAPK signaling governs ISC stemness as well as their differentiation into TA cells (48). Of the many MAPK signaling pathways, the Ras/Raf/MEK/ERK system is the best characterized which culminates in the terminal phosphorylation, and thus activation, of the MAPKs ERK1 and ERK2 (Figure 2B). MAPK/ERK signaling is potentiated by the Src homology 2 (SH2) phosphatase 2 (SHP2), a ubiquitously expressed cytoplasmic phosphotyrosine (pY) phosphatase whose target substrate is the EGF receptor (EGFR) which is a transmembrane receptor tyrosine kinase (RTK) (49). Deletion of SHP2 in IECs results in a decreased ERK phosphorylation (50) whereas its activation confers resistance to dextran sulfate sodium (DSS)-induced colitis and *Citrobactoer rodentium* (*C. rodentium*) infection through the MAPK/ERK pathway (51). At present, the extent to which AhR activation works in tandem with SHP2-MAPK/ERK signaling to promote intestinal homeostasis remains largely unknown; however, its role in potentiating MAPK/ERK signals must be highlighted. Independent of SHP2, AhR activation by the tryptophan derivative 6-formylindolo (3, 2-b) carbazole (FICZ) has indeed shown to ameliorate DSS-induced colitis and exclusively promote the MAPK/ERK-dependent differentiation of GCs (52). Importantly, this selectivity for GCs occurs in parallel with a suppression of Notch signals as indicated by a down-regulation of the Notch intracellular domain (NICD) which is released upon Notch activation (Figure 1B). Like the Wnt/β-catenin pathway, Notch signaling has a profound effect on intestinal development as it regulates ISC stemness and epithelial cell fate (53, 54). In this regard, Notch signaling suppresses the differentiation of GCs and its actions may therefore be countered by the activation ERK as previously described (51). Taken together, these findings suggest that AhR-MAPK/ERK signaling promotes intestinal homeostasis by selecting for the differentiation of GCs.

## The Contribution of AhR Ligands to ISC Homeostasis

### Tryptophan Metabolites

Tryptophan is an essential amino acid and is a precursor for several bioactive molecules, especially serotonin; however, only a small percentage of tryptophan is metabolized into serotonin. Instead, ~ 95% of tryptophan is metabolized into kynurenine (KN) which plays a critical role in cellular energy production following its eventual conversion into nicotinamide adenine dinucleotide (NAD+) through the kynurenine pathway (KP) (55, 56). What remains of the KN pool under physiological conditions is converted into kynurenic acid (KA) (56) and both metabolites are potent AhR ligands (57, 58). Interestingly, KN may regulate epithelial cell fate through the AhR. Of note are recent findings demonstrating that both tryptophan and KN promote GC differentiation in HT-29 cells as determined by Muc2 gene expression (59). Analyses confirmed that both inhibition of KN synthesis by 1-Methlytryptophan (1-MT) and inhibition of AhR signaling by its antagonist α-naphthoflavone suppresses Muc2 gene expression, suggesting a loose connection between AhR activation and KN synthesis in the production of GCs. Importantly however, while KN was shown to increase the protein expression of β-catenin relative to NICD (Wnt vs. Notch signals), these effects were dependent on the media in which the cells were grown (i.e., DMEM vs. RPMI) which can vary in amino acid and glucose content (59). Interestingly, an early report by Park et al. has indicated that AhR is highly expressed in LGR5+ stem cells in the small intestine and that administration of its potent ligand FICZ, a tryptophan derivative generated by ultraviolet B irradiation (60), inhibits the development of intestinal organoids in a concentration-dependent manner *in vitro* as indicated by significant reduction in absolute numbers of organoids and slightly reduces Paneth cells in the small intestine with a concomitant reduction in crypt length and a reduction in colonic crypt length *in vivo* (61). It was also found that FICZ reduced the protein expression of active β-catenin in organoids derived from small intestinal crypts (perhaps due to a loss of morphogenetic factors produced from crypt Paneth cells) though increased the gene expression of the transcription factor *ATOH1*, which promotes the differentiation of secretory lineages from ISCs, as well as altered the gene expression of other morphogenetic pathway markers (61). Though no changes in GC number were observed following FICZ administration, the observed increase of *ATOH1* expression highlights the putative role of FICZ in promoting the differentiation of GCs as previously shown (52). In all, these findings suggest that KN promotes the differentiation of GCs in tandem with AhR activation but that these actions may be dependent on additional local factors such as other amino acids. In addition, FICZ modulates multiple morphogenetic pathways and its effect on epithelial cell fate may be consistent with KN in promoting the differentiation of GCs but the suppression of Paneth cells and thus the extent to which FICZ modulates differentiation of secretory lineages warrants further investigation. Further, the selective reduction in Paneth cell number may reflect region-specific effects of FICZ on epithelial cell fate.

### Microbiota-Derived

The gut microbiota encompasses a diverse array of microbial taxa that colonize the full length of the GI tract, consisting of approximately 3.8 × 10^13^ cells in total (62). The majority of the gut microbiota is harbored in the colon and modulates its host's physiology by the production of microbiota-derived metabolites that act upon multiple organ systems through various “host-microbe metabolic axes” (63). These metabolites include (but are not limited to) tryptophan catabolites and short-chain fatty acids (SCFAs) originating from the bacterial fermentation of dietary protein and soluble fiber (64, 65). These metabolites serve as AhR ligands of varying affinities and may affect ISC stemness.

#### Tryptophan Catabolites

As mentioned above, much of dietary tryptophan is metabolized into KN through the KP as the majority of ingested protein is digested and absorbed in the small intestine (66). Depending on total intake however, excess protein and amino acids (6–18 g/day) may reach the colon and become accessible to the resident gut microbiota (67). While there are bacteria that specialize in the proteolytic fermentation of dietary protein, the degradation of tryptophan appears to be a ubiquitous function shared among several bacterial species that reside throughout the GI tract (65, 68). Most notably is the ability of the gut microbiota to convert tryptophan into indole and indole derivatives via the enzyme tryptophanase (TnaA) (69, 70). To date, research indicates that a variety of both Gram-positive and -negative bacteria are capable of producing large amounts of indole and consequently, that indole acts as a significant signaling molecule within microbial communities having been implicated in the control of diverse aspects of bacterial physiology as reviewed elsewhere (71). Given its importance in shaping the ecological landscape and physiology of the gut microbiota, bacteria-derived indole and its derivatives have a significant impact on host gut physiology and health. While several derivatives of indole exist, here we focus on the AhR ligands indole-3-aldehyde (IALD) and indole-3-propionic acid (IPA) and their prospective contribution to ISC stemness as these two ligands have shown to directly impact ISC stemness to date.

Among the many aspects of immune development that AhR signaling plays a role in, notably is its impact on innate lymphoid cells (ILCs). ILCs are a heterogenous population of immune cells that are non-T and non-B lymphocytes which lack antigen-specific receptors and are hence activated through cytokine signaling (72). ILCs have distinct groups that express transcription factors and produce signature cytokines including group 3 ILCs (ILC3s), which release interleukin (IL)-22 upon AhR activation (73). This AhR-IL-22 axis expressed in ILC3s is critical for the maintenance of intestinal homeostasis as AhR deficiency in RORγt^+^ ILCs increases susceptibility to *C. rodentium* infection due to a lack of IL-22 production (74). Likewise, AhR deficiency in mice causes an increase in Th17 cells and an expansion of commensal segmented filamentous bacteria (SFB) due to a concomitant reduction in IL-22 (75). Further, haplodeficiency of RORγt with genetic ablation of AhR spontaneously induces colitis indicating the importance of RORγt in maintaining the ILC3 compartment and subsequent IL-22 production in tandem with AhR activation (75). Additional findings confirm that treatment with IL-22 increases ISC stemness both *in vivo* and *ex vivo* as well as reduces intestinal pathologies associated with graft-versus-host disease (76). In this same study, it was also found that STAT3 activation was crucial for both organoid formation and IL-22 mediated epithelial regeneration highlighting the importance of JAK/STAT signaling in ISC stemness. While evidence also illustrates a Notch-AhR-IL-22 axis which regulates colon tissue homeostasis through the development of IL-22 producing ILCs (77–79), an earlier report has shown that IALD produced primarily from *Lactobacillus reuteri* (*L. reuteri*) increases the production of IL-22 in indoleamine 2,3-dioxygensase 1 (IDO1) deficient mice, conferring antifungal resistance and mucosal protection when challenged with *Candida albicans* (*C. albicans*) or DSS (80). As expected, these beneficial effects were not observed in AhR-deficient mice emphasizing the AhR-dependent release of IL-22 (80). In addition, a more recent study explored the protective effect of *L. reuteri* on the integrity of the gut mucosa in an attempt to elucidate the therapeutic benefits of Lactobacilli often found in yogurt (81). The authors reported that *L. reuteri* upregulated IL-22 production and stimulated ISC regeneration (as indicated by an increase in LGR5+ stained cells) in both organoid/LPL co-cultures and in mice which was also observed with IALD administration. Lastly and similar to the findings of Lindemans et al. (76), the secretion of IL-22 by LPLs stimulated with *L. reuteri* or IALD increased the phosphorylation of STAT3 both *in vivo* and *ex vivo*. Together, these findings suggest that IALD derived from Lactobacilli plays a pivotal role in the production of IL-22 within AhR-expressing immune cells and that through the AhR-IL-22 axis, promotes ISC regeneration and epithelial restitution which is dependent on STAT3 activation.

IL-10 is a potent anti-inflammatory cytokine whose significance is well established in IBD. This important cytokine signals through the IL-10 receptor ligand-binding subunit (IL-10R1) and is induced during inflammation to suppress the production of proinflammatory mediators in IECs (82). To date, studies indicate that IL-10 regulates mucin biosynthesis in GCs and that IL-10 is critical for Paneth cell development and function (83, 84). While the direct effect of IL-10 on IEC function and development is less explored, these findings suggest that IL-10 may have an influence on secretory epithelial cells. Nevertheless, a recent report has shown that both IALD and IPA induce IL-10R1 expression *in vitro* and that this induction requires AhR signaling as the ablation of its dimeric partner ARNT prevented the indole-dependent induction of IL-10R1 (85). Moreover, only wild-type *Escherichia coli (E. coli)* were able to generate IALD and IPA, and thus induce epithelial IL-10R1. Collectively, these results indicate a putative role of IL-10 signaling in secretory epithelial cell function and development, and that the microbiota-derived indole derivates IPA and IALD augment the therapeutic effects of IL-10 via AhR signals in the preservation of mucosal homeostasis.

#### Short-Chain Fatty Acids

SCFAs are one of the major end products of microbial fermentation and are formed from carbohydrate, protein and glycoprotein precursors by anaerobic bacteria (86). Principal SCFAs are acetate, propionate and butyrate in which all are important sources of carbon and energy for host tissues (87). These organic acids are absorbed through the gut mucosa and can modulate host energy homeostasis through interactions between chemosensory enteroendocrine cells (87, 88). Interestingly, butyrate is a critical energy source for colonocytes (89) and exhibits therapeutic effects like that of other AhR agonists including induction of Treg cells, anti-inflammatory responses as well as the induction of IL-22 (90–93). While recent data show that all three SCFAs enhance AhR responsiveness *in vitro* primarily as histone deacetylase (HDAC) inhibitors (94), additional findings demonstrate that butyrate can activate AhR signaling independent of its role as an HADC inhibitor suggesting that it is a direct AhR ligand as well (95). As recently reviewed (96), studies that have investigated the effect of butyrate on ISCs are discrepant, however. For instance, as butyrate is a primary energy source for colonocytes (89), it can facilitate ISC proliferation through gluconeogenesis (97) and improved microcirculation by dilating colonic resistance arteries (98). In contrast, butyrate has shown to suppress colonic stem cell proliferation by HDAC inhibition and Foxo3 regulation, a transcription factor that governs cell proliferation and longevity (99). While disagreements remain, studies to date overall posit that butyrate regulates ISC proliferation in the colon and controls the differentiation of GCs. Still however, the extent to which butyrate regulates ISC stemness via AhR activation remains elusive.

### Plant-Derived

Given the ubiquitous influence of AhR signaling in the maintenance of barrier surfaces and its ability to ligate numerous ligands, the efficacy of natural AhR ligands in the treatment of inflammatory disorders in murine models has been extensively explored. Of note are the phytochemicals quercetin, resveratrol and indole-3-carbinole (I3C). At present, there are no studies that have examined the effects of these flavonoids in IBD patients but the therapeutic aspects thereof have been extensively studied due to their potent antioxidant and anti-inflammatory properties (100). While each indeed has potent therapeutic effects in the treatment of experimental IBD (101), recent evidence suggests that these effects extend beyond modulation of immune responses and inflammation in which maintenance of ISC stemness may also be a benefit.

Quercetin is an abundant polyphenol found in many natural foods including fruits, vegetables, and nuts (102). Like quercetin, resveratrol is a polyphenol best known to be enriched in the skins and seeds of red grapes used to make red wine (103). Due to their low affinity, both are corroborated to be indirect AhR ligands and control AhR responsiveness by inhibiting the actions of CYP1A1 which prevents the metabolic turnover of the potent AhR agonist FICZ (104). As shown above, multiple signaling pathways, including MAPKs and canonical Wnt/β-catenin cascades, regulate cellular turnover of the intestinal epithelium. Expectedly, oncogenic mutations inducing the hyperactivation of both pathways perturb intestinal homeostasis and result in intestinal malignancies. Of note are the oncogenic mutations of K-Ras (KRAS) within the EGFR-MAPK/ERK pathway which has shown to be involved in CRC development (105, 106). Resveratrol has been found to possess a broad-spectrum of health benefits including anti-cancer activities (107) and findings by Saud et al. specify that resveratrol acts directly to suppress KRAS expression (108). Using a conditional knockout model of APC in mice supplemented with resveratrol, the authors determined that resveratrol inhibits tumor growth and proliferation which is accompanied by a reduction in LGR5, KRAS and nuclear β-catenin expression. Interestingly, mRNA levels of KRAS did not change with resveratrol but instead, an 80% increase in the expression of the miRNA miR-96 was observed. As miR-96 has shown to regulate the translation of KRAS mRNA (109, 110), the authors concluded that the mechanism through which resveratrol confers its therapeutic effects is the post-translational modification of KRAS by miRNAs. Similarly, recent findings of Damiano and colleagues suggest that the therapeutic effects of quercetin are also enacted via the MAPK/ERK pathway particularly as it relates to GC function (111). In human intestinal GC-like LS174T and Caco-2 cells, the authors observed a significant increase in MUC2 and MUC5AC expression in both cell lines following exposure to quercetin and that these effects were dependent on the induction of both MAPK/ERK and protein kinase C alpha (PKCα) signals. PKC is a family of lipid-sensitive serine/threonine protein kinases that regulate various cellular functions including cell proliferation, differentiation, migration, adhesion and apoptosis (112). Importantly, PKCα activity is a strong agonist of ERK signaling via Ras activation and works in parallel to regulate cell cycle withdrawal in IECs (113). Taken together, these studies suggest that resveratrol and quercetin promote intestinal homeostasis through opposing directions of the same signaling cascade; resveratrol regulates cell proliferation by inhibiting Wnt and MAPK/ERK signals (via the suppression of KRAS) whereas quercetin modulates the biosynthesis of mucins in intestinal GCs *via* the activation of MAPK/ERK and PKCα signals. Regarding AhR signaling, quercetin may confer its effects on intestinal GCs indirectly by allowing the FICZ-AhR-MAPK/ERK axis discussed above to ensue whereas resveratrol may exert its therapeutic effects via the induction of miR-96 which has shown to be regulated by the AhR in the lung (114).

I3C is a breakdown product of glucobrassicin, a sulfur-containing compound that is rich in cruciferous vegetables such as broccoli and cabbage and is converted primarily into 3,3′-diindolylmethane (DIM) due to the acidic environment in the stomach upon digestion (115). I3C has shown much promise in the treatment of IBD as we have recently demonstrated that it prevents colitis via the induction of IL-22 (116), further highlighting the importance of the AhR-IL-22 axis in intestinal homeostasis. With regard to ISC stemness, a recent report by Park et al. further associates I3C-AhR induction with both Wnt and Notch signals in the regulation of GC differentiation (117). Similar to their earlier report using FICZ (61), administration of I3C by oral gavage inhibited the development of intestinal organoids in an AhR-dependent manner as indicated by a decrease in the proliferation of both ISCs and TA cells. RNA expression analyses of lineage specific genes in cultured organoids further concluded that I3C directly impacts the development of GCs, Paneth cells and enterocytes such that I3C increases MUC2 and lysozyme expression but decreases intestinal alkaline phosphatase (IAP) expression. In addition, and in contrast to their previous report, GCs were increased in I3C-treated mice. Given the preferential increase of genes related to secretory epithelial cell types, additional analyses confirmed that I3C indeed potentiates Wnt but suppresses Notch signals as evidenced by an increase in β-catenin and a decrease in Notch protein expression as well as in *HES1* RNA expression, a transcription factor activated downstream of Notch signaling which suppresses ATOH1. While these findings suggest that I3C potentiates Wnt signaling, it may be context dependent as Metidji et al. have demonstrated that dietary I3C tempers Wnt hyperactivity in *Villin*^*Cre*^*R26*^*LSL*−*Cyp*1*a*1^ mice co-challenged with azoxymethane (AOM)/DSS by enhancing the expression of ZNRF3 and RNF43 (9). This discrepancy might be due to mode of administration of I3C as purified diets may potentiate differential effects on intestinal health in comparison to normal chow (118). In sum, these findings posit that I3C plays a direct role in the development of ISCs via the AhR perhaps in a context-specific manner to maintain intestinal homeostasis and indicate that this regulation is likely mediated by both Wnt and Notch signals.

## Conclusions

The ISC niche is complex and is the epicenter from which all intestinal epithelial cells arise. The fate of these stem cells and the function of their differentiated progeny are driven by varying local factors whose actions are coordinated through numerous signaling cascades that blend to govern ISC stemness. To add to this complexity, evidence reported herein highlights the extensive integration of AhR activation by various AhR ligands in the regulation of such pathways associated with ISC stemness. What proves challenging moving forward is addressing the promiscuous nature of AhR signaling itself. To remedy this, animal studies that investigate the effects of AhR deficiency in a cell-specific manner together with global ablation could provide more insight into the exact mechanisms through which the AhR exerts its effects. In addition, mode of dietary ligand administration (refined diets vs. intraperitoneal injection vs. oral gavage) should be strongly considered as each method could differentially affect experimental outcomes. Altogether, while much of the responses from AhR activation are context- and cell-dependent, the present findings illustrate the ubiquitous effects of AhR signaling in the maintenance of the ISC niche. What remains to be explored further is the extent to which both the mucosal immune system and the induction of molecular cascades in epithelial cells work in tandem with the AhR to regulate ISC stemness and epithelial cell fate.

## Author Contributions

PW: wrote the manuscript and designed the figures. MN: provided extensive input regarding the focus and organization of the manuscript. PN: provided extensive editing and additional content to the manuscript. All authors contributed to the article and approved the submitted version.

## Conflict of Interest

The authors declare that the research was conducted in the absence of any commercial or financial relationships that could be construed as a potential conflict of interest.
